# Subperiosteal facelift: a 5-year experience

**DOI:** 10.1016/S1808-8694(15)31014-4

**Published:** 2015-10-19

**Authors:** Lucas Gomes Patrocínio, José Antônio Patrocínio, Hugo Gonçalves Couto, Hélio Muniz de Souza, Paulo Márcio Coelho Carvalho

**Affiliations:** a MD. Otorhinolaryngology Resident at the Medical School of the Federal University of Uberlândia.; b Full Professor. Head of the Otorhinolaryngology Department of the Medical School of the Federal University of Uberlândia.; c MD. Otorhinolaryngology Resident at the Medical School of the Federal University of Uberlândia.; d MD. Otorhinolaryngology Resident at the Medical School of the Federal University of Uberlândia.; e MD. Otorhinolaryngology Resident at the Medical School of the Federal University of Uberlândia. Otorhinolaryngology Department of the Medical School of the Federal University of Uberlândia, Uberlândia, Minas Gerais, Brazil.

**Keywords:** Rhytidoplasty, Subperiosteal, Plastic Surgery, Endoscopy

## Abstract

In classic rhytidectomy, there is little improvement in the center portion of the face. Aesthetic correction of malar prominence ptosis, accentuated nasolabial line, and jawl line, in most of the cases, require different approaches, such as the subperiosteal facelift.

**Aim:**

to show the cases and to evaluate the results and complications of subperiosteal facelift in the our service.

**Patients and Methods:**

From January of 2001 to December of 2005, 25 patients, ranging from 44 to 60 years, 24 females, were submitted to subperiosteal facelift. Results and complications were retrospectively appraised.

**Results:**

Of these, 20 presented satisfactory results, 4 presented aesthetic deficits noticed both by the patients and by the surgeon, and 1 presented aesthetic deficit needing revision surgery. All the patients presented improvement of nasolabial line, malar prominence and better definition of the jawl line. Revision surgery was necessary in one patient that referred little improvement. Four patients presented skin retraction in malar area due to the suspension sutures. A patient presented transitory paralysis of the front branch of the facial nerve.

**Conclusion:**

Subperiosteal facelift with temporal access has shown satisfactory results in the great majority of the cases.

## INTRODUCTION

In the last century, Medicine has enjoyed spectacular progress in the most diverse areas, specially as far as cosmetic surgery is concerned, allowing individuals to reach advanced ages not only fit and in good health, but also having a more youthful appearance. Gravity together with sun light exposure and the loss of skin elasticity because of the natural human aging process result in different levels of wrinkles on the face. Many procedures have been proposed in order to correct facial wrinkles, and today it is agreed that the best surgical option to correct such problem is rhytidoplasty^1^, ^2^.

Rhytidoplasty, or facial lifting, is today one of the most sought upon procedures by patients above 40 years of age, aiming at rejuvenating the face. A number of facial wrinkles removal techniques have been described along the years.

The first generation of these was the skin rhytidoplasty, in which only the skin is removed. The second generation came with the description of the SMAS (subcutaneous muscle-aponeurotic system). In such modality, the surgeon treats the SMAS (plication, suture, partial sectioning, etc.) aiming at increasing procedure duration. The third generation came exactly with the attempt to reach the nasogenal groove, which so far was not altered by the other techniques. Deep plane rhytidoplasty allows for a deep SMAS dissection. However, its use is being increasingly abandoned because of the risk of damaging facial nerve branches^2^, ^4^.

In order to act on this “untouchable” oval facial center, Psillakis^3^ and, soon after, many other authors^5^, ^6^, ^7^, ^8^, ^9^ described the subperiosteal rhytidoplasty. In the present study we wish do demonstrate a series of patients and assess results and complications accruing from the subperiosteal rhytidoplasty through a temporal incision in our Department of Otorhinolaryngology of the Federal University of Uberlândia.

## PATIENTS AND METHODS

From January 2001 to December, 2005, we carried out 25 subperiosteal rhytidoplasties at the Department of Otorhinolaryngology of the Federal University of Uberlândia. Patients were between 44 and 60 years of age, and 24 were females.

All patients were examined and went through a strict selection process in order to undergo this procedure. We selected only those patients with relevant nasolabial groove, malar prominence ptosis and poor jaw line definition. The procedure was contraindicated in patients with increased jaw line ptosis, satisfactory malar prominence and not pronounced nasolabial groove.

**Figure 1:**
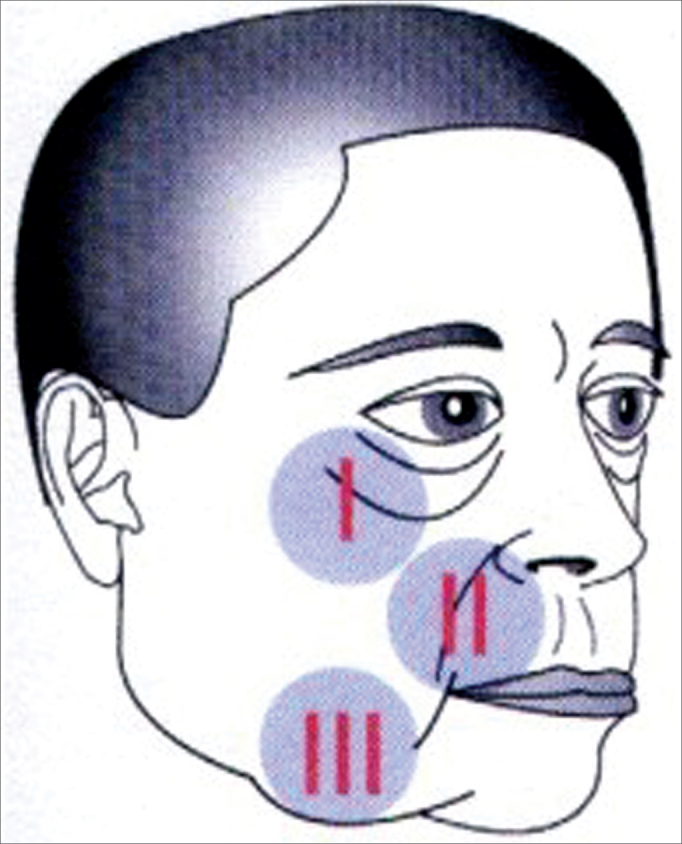
Drawing showing the three regions assessed during the subperiosteal rhytidoplasty: I) Infra-orbit malar complex, II) Nasolabial groove; III) Jaw line.

Patients returned at 7, 30, 60 and 180 days of postop and were reassessed as to possible complications. In the last visit the patients were questioned as to their satisfaction towards the procedure and the final cosmetic result was judged by the medical team. The following regions were assessed: I) Malar prominence; II) Nasolabial groove; III) Jaw line (Figure 1).

All the patients signed an informed consent form in which they were educated about the surgery, in agreement with resolution # 016/2005 of the Research Ethics Committee.

### Surgical Approach

Anesthesia was carried out by sedation (fentanyl and midazolam) and local anesthesia (2% lidocaine with 1:100.000 adrenalin).

The surgeon was positioned upwind to the patient's head, with the video monitor to his left. The patient was prepared with a good facial and scalp cleaning and disinfection. The hair was braided and fixed by Micropore tape, and the incision sites were marked. After that, a 5cm lateral incision was carried out in the coronal direction, after hair removal (3cm away from the hair line), 2cm above and below the temporal line in both sides. For the endoscopic frontoplasty cases associated, incisions, detachments and sutures were carried out in this step, as previously described^10^.

On the temporal region, laterally, the deep temporal fascia was exposed and the detachment went all the way down to the medial third of the zygomatic arch and orbit border. On this site, the zygoma periosteum was incised and the subperiosteal detachment was extended to the malar and to the nasolabial and gengivolabial grooves.

All the detached area released the insertions of the orbicular, zygomatic major and minor and other muscles, eyelids, external canthal and Lockwood ligaments, parotid fascia inferiorly and temporal fascia superiorly.

**Figure 2:**
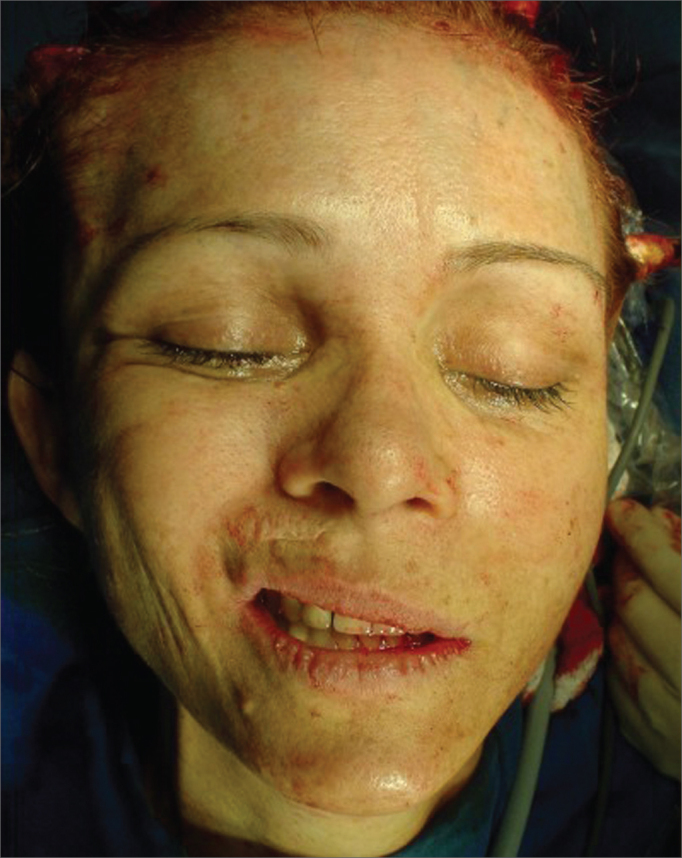
Photography showing right-side hemiface middle third elevation during the procedure.

The zygoma periosteum and that of the parotid fascia were fixed to the temporal muscle fascia by means of three suspension sutures with Ethibond 2-0. Systematization of the facial middle third lift was carried out by three main points: Bichat's ball, malar fat and eye suborbicular fat (SOOF). These three previously marked points were lifted by Ethibond 2-0 by means of handle sutures made by Reverdin needle and attached to the temporal fascia, the Bichat ball medially and the SOOF laterally. Such fixation stretches the zygomatic muscles and the soft tissue of the cheeks, thus correcting jugal region drop, enhancing the nasolabial groove. The zygomatic area is also well modeled because the zygomatic muscle insertions are reinserted in a higher position (Figure 2).

A Penrose tube was left for 24 to 48 hours and the skin was sutured by Mononylon 4-0. We made a compressive dressing on the forehead that stayed on for 7 days, when the stitches were removed. A compressive bandage was kept on during the first 6 to 10 hours of postop.

## RESULTS

The final surgical subjective assessment depends on the viewpoints of both patient and surgeon, that in some cases can be very different. Of the 25 patients, 20 presented with satisfactory results, 4 had cosmetic deficits, noticed by both patient and surgeon, and one had a cosmetic deficit and needed revisional surgery.

**Figure 3:**
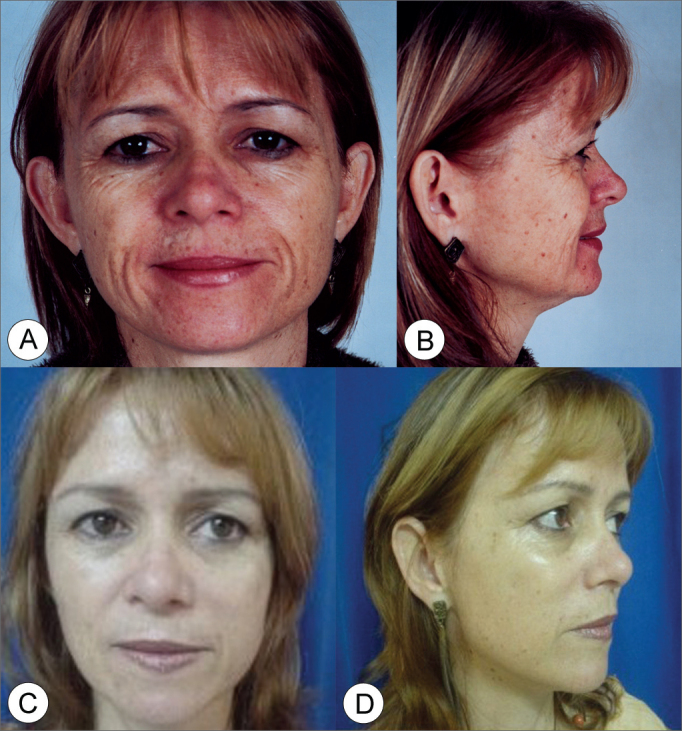
Frontal view (A) and side view (B) of a 47 year old female patient with enlarged nasolabial groove, relevant drop of malar prominence and mild jaw line ptosis. (C and D) Subperiosteal rhytidoplasty postoperative image (3 years) depicting malar prominence elevation, reduction in the nasolabial groove and jaw line improvement.

**Figure 4:**
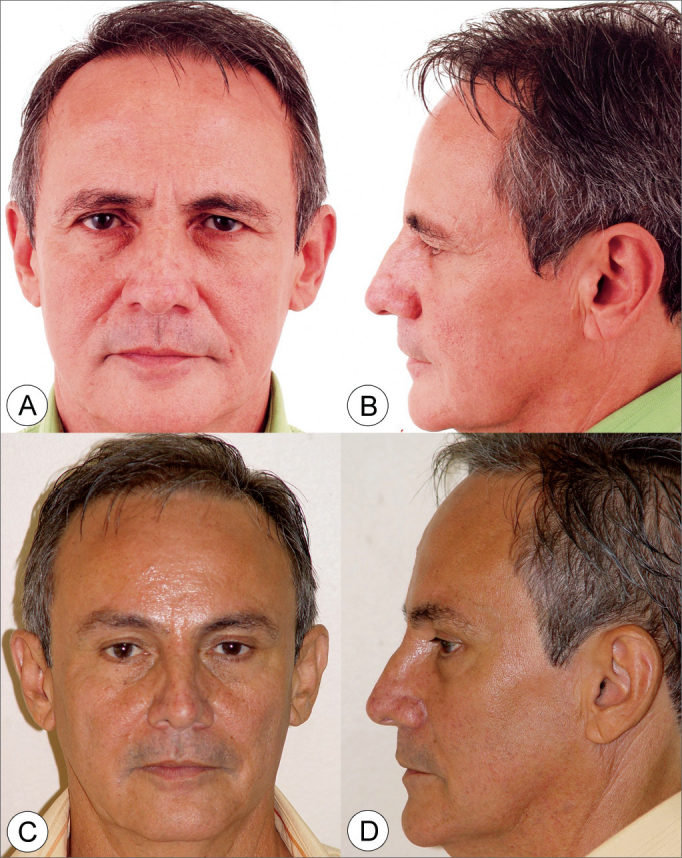
Frontal view (A) and side view (B) of a 52 year old male patient with enlarged nasolabial groove, relevant drop of malar prominence and moderate jaw line ptosis. (C and D) Subperiosteal rhytidoplasty and endoscopic frontoplasty postoperative image (2 years) depicting malar prominence elevation, reduction in the nasolabial groove and jaw line improvement.

**Figure 5:**
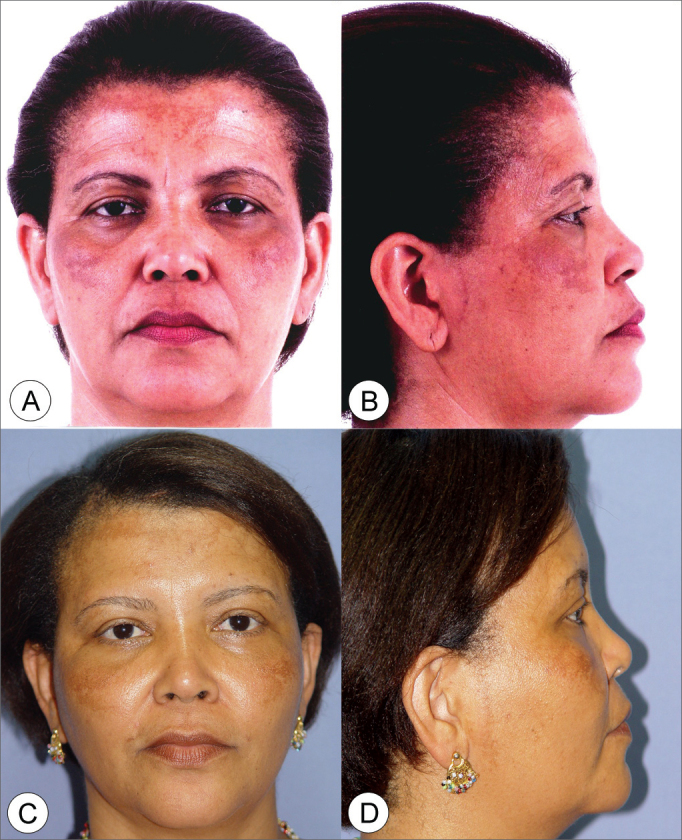
Frontal view (A) and side view (B) of a 45 year old female patient with enlarged nasolabial groove, moderate drop of malar prominence and moderate jaw line ptosis. (C and D) Subperiosteal rhytidoplasty and endoscopic frontoplasty postoperative image (1 year) depicting malar prominence elevation, reduction in the nasolabial groove and jaw line improvement.

**Figure 6:**
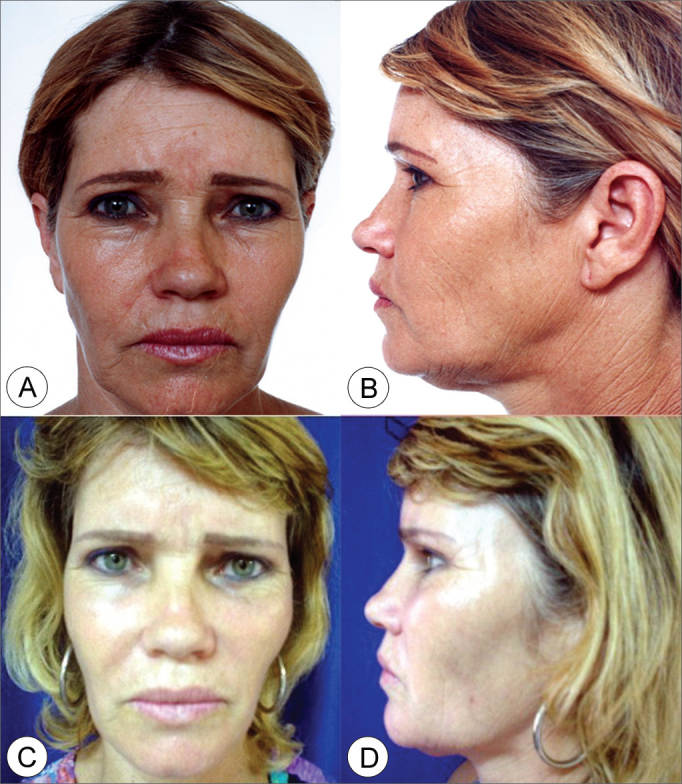
Frontal view (A) and side view (B) of a 51 year old female patient with moderately enlarged nasolabial groove, moderate drop of malar prominence and moderate jaw line ptosis. (C and D) Subperiosteal rhytidoplasty postoperative image (4 years) depicting malar prominence elevation, reduction in the nasolabial groove and jaw line improvement.

On photographic study, all patients presented improvements in their nasolabial groove, malar prominence and better jaw line definition (Figure 3, Figure 4, Figure 5, Figure 6). Empirically, of these three points analyzed, the jaw line is the one with the least improvement. Revisional surgery was needed in one patient that did not feel the surgery was successful in matching his expectations.

There were complications in 6 cases. One patient had a mild serohematoma that needed to be drained. Four patients presented skin retraction in the malar region caused by the lifting sutures. One patient had transitional paralysis of the facial nerve frontal branch. There were no other complications such as cheloid formation, hypo or hyperpigmentation, nor granuloma in the incision.

## DISCUSSION

Subperiosteal rhytidoplasty started in 1982 with Psillakis^2^, who did a coronal and pre-auricular incision all the way to the tragus. It was also described by Santana in 1984^5^ and Tessier in 1989^6^. It was greatly improved with the use of endoscopes as of 1991 by Keller^7^ and Ramirez^8^. Hester et al.^9^ described the technique through blepharoplasty.

Subperiosteal rhytidoplasty has attracted the attention of many authors, since it aims at raising the eyebrows, eyelid lateral corner, forehead, glabella, cheeks and nasolabial groove, reaching the middle portion of the face. This technique includes less incision, use of endoscope, better fixation - specially of the cheeks, less skin incision, allows for more ancillary procedures, repositioning of the Bichat ball, and jaw treatment^7^, ^8^.

Subperiosteal rhytidoplasty is indicated for patients with significant aging and ptosis of the oval center of the face, infraorbitary tear-shaped deformity, sclera show in severe malar pockets, in cases of past facial fractures, when there is the need for simultaneous resurfacing, in cases of facial implants that need to be changed, when there is a need for soft tissue augmentation with fat tissue and even in smokers (subperiosteal dissection preserves blood supply).

In our series, all the patients presented malar prominence elevation, reduction in nasolabial groove and jaw line improvement. We have empirically noticed that this region is the one that benefits the least from this technique. Therefore, when there is marked jaw line ptosis, a conventional rhytidoplasty is the approach of choice, together with SMAS and platysma treatment^2^.

In recent years, the subperiosteal rhytidoplasty technique has been increasingly discussed and progressively accepted by facial surgeons. Notwithstanding, there still is much controversy at to incision type, suturing techniques, postoperative, result duration, complications, etc. Freeman^11^ believes the endoscopic elevation of the facial middle third is as efficient as the open procedure as far as rejuvenation is concerned, bearing the advantage of dismissing the pre-auricular incision, besides allowing malar area action, with skin incision in the lower eyelid - bearing lower risk of causing ectropium. This author, in particular, indicates this approach for patients with scar deformities in the hemiface, in heavy smokers and lip corner drop cases. This method can, actually, be used in any patient who wishes to improve lip corner appearance.

Other authors such as De la Plaza and De la Cruz^12^ believe the supraperiosteal approach favors a more comfortable and less traumatic dissection. They do not agree that the subperiosteal dissection can manage a selective and effective tetrastructure migration (orbicular muscle, temporo-parietal fascia, SMAS and frontal muscle) due to a limitation brought about by the periosteum rigidity and lack of elasticity.

Differently from that, Ramirez^8^, ^13^ believes that by subperiosteal rhytidoplasty, the maxillary area may be repositioned upwards. He adds that being the periosteum without elasticity, this allows it to be released and moved in block with the structures. He says the endoscopic approach has been surprisingly high, it is less traumatic and able to remodel the oval facial center in a way that would be much more difficult, dangerous and even impossible by any other method. The also states that facial cosmetic surgery has enjoyed great progress, changing access ways: this trend towards deeper plane dissection, the growing use of endoscopes reducing scars, increasingly stressing the oval center of the face, besides ancillary procedures of refinement such as laser, fat grafting, and aloplastic implants, etc. Ramirez^8^, ^13^ thinks the results of this technique are significant better: less facial edema, faster recovery, less eyelid problems, directive and effective malar elevation and a balanced facial appearance.

In order to achieve such elevation, in our department, we perform temporal incision after hair removal^10^ and we section the periosteum as lower as possible and then we may, through traction by Ethibond 2-0 wires, raise the tetrastructure. De la Plaza and De la Cruz prefer the bicoronal access incision, considering this an excellent via for men, since it does not leave visible scars. Keller et al.^14^ prefer to dissect over the malar bone through an inferior blepharoplasty incision; however, they admit that the access through the gengivobuccal groove may be also used.

Krastinova-Lolov^15^ described the mask-lift as a revolutionary and different access, that normalized, rejuvenates and embellishes the face throughout a subperiosteal lifting. Differently from our approach, the mask-lift is performed through a bicoronal incision. As potential complications we have chemosis or conjunctiva edema, temporal or parietal hematoma, paralysis of the facial nerve frontal branch. According to Krastinova-Lolov, the final surgery result may take up to a year to be reached, however it lasts longer and is more natural looking.

As far as complications are concerned, there were 2 light events (serohematoma and transitory frontal paralysis) that did not bring clinical complications to the patients. There was malar retraction at the suspension suture site in four patients, in whom it was necessary to perform a mild procedure to shift skin and subcutaneous tissue in order to solve the problem. This is a complication that is inherent to the learning curve of any surgical technique that, with time and training ceased to occur.

## CONCLUSIONS

We conclude that subperiosteal rhytidoplasty by temporal approach, is a technique that produces satisfactory cosmetic results in most of the cases, causing malar prominence elevation, nasolabial groove improvement and jaw line improvement.
